# Association of symptoms of attention-deficit/hyperactivity disorder with symptoms of excessive exercising in an adult general population sample

**DOI:** 10.1186/s12888-014-0250-7

**Published:** 2014-09-12

**Authors:** Nikolas AA Berger, Astrid Müller, Elmar Brähler, Alexandra Philipsen, Martina de Zwaan

**Affiliations:** Department of Psychosomatic Medicine and Psychotherapy, Hannover Medical School, Carl-Neuberg-Straße 1, 30625 Hannover, Germany; Department of Medical Psychology and Medical Sociology, University of Leipzig Medical Center, Leipzig, Germany; Department of Psychosomatic Medicine and Psychotherapy, University Medical Center Mainz, Mainz, Germany; Department of Psychiatry and Psychotherapy, University Medical Center Freiburg, Freiburg, Germany

**Keywords:** Attention-deficit/hyperactivity disorder, Excessive exercising, Physical activity, Exercise, Adult, General population

## Abstract

**Background:**

An increasing number of studies suggest that physical activity can alleviate symptoms of ADHD in children. In adults there are currently insufficient data available on this subject. Interestingly, ADHD and forms of excessive exercising have both been shown to occur more frequently in adult athletes. The aim of the present study was to empirically investigate the association of ADHD and excessive exercising in the adult general population.

**Methods:**

For diagnosis of adult and childhood ADHD a large representative sample of the German general population (n = 1,615) completed a retrospective assessment of childhood ADHD and a self-report assessment of adult ADHD. Excessive exercising as well as putative mediating variables such as eating related psychopathology, depression, and anxiety were assessed using standardized self-rating instruments.

**Results:**

Individuals with childhood only ADHD had a significantly higher frequency of excessive exercising (9.0%) than individuals without ADHD (2.7%). Excessive exercising was significantly associated with childhood only ADHD compared to no ADHD with an odds ratio of 3.239 even after controlling for socio-demographic variables, BMI, eating related and general psychopathology.

**Conclusions:**

Our data show that excessive exercising is significantly overrepresented in individuals in which ADHD symptoms in childhood have not persisted into adulthood. We thus hypothesize that a subgroup of individuals might suppress ADHD symptoms by excessive sporting activities. Although in healthy adults physical activity has been associated with immediate and long term improvements in cognitive functioning, studies empirically investigating associations between the effects of physical activity and adult ADHD are rare. Further studies are warranted to explore the potential role of physical activity in the treatment of ADHD in adults.

## Background

Attention-deficit/hyperactivity disorder (ADHD) is a common neurodevelopmental disease, clinically defined by its characteristic core symptoms of inattention, and/or hyperactivity and impulsive behavior [1]. The symptoms usually cause functional impairment in social, occupational and academic terms [2]. Structural and functional deficits of attention and executive function processing brain networks have been shown in children and adults with ADHD [3,4]. Furthermore ADHD has been associated with reduced levels of brain-derived neurotrophic factor (BNDF), central for normal brain development and vital to the survival and differentiation of neurons and synapses in brain areas essential for memory and learning [5].

Traditionally, ADHD has been seen as a self-limiting childhood disease, but recent research has revealed that in most cases major symptoms of the disease persist into adulthood [6,7]. It is estimated that ADHD has a prevalence of 5.3% among children and adolescents and 2-4% in the adult general population [1,8,9]. In a 10-year follow up study, Biederman et al. [10] found that young adults diagnosed with ADHD were at high risk for a wide variety of comorbid psychiatric disorders including increased rates of antisocial, mood, anxiety and addictive disorders. Likewise eating disorders have been associated with ADHD [11].

There is a growing body of evidence pointing towards the positive effects of physical activity (PA) on ADHD symptoms and executive function in children [12,13]. Table 1 gives a literature overview on effects of regular physical activity on ADHD symptoms in children. We included studies with moderate or vigorous intensity PA applied continuously over several weeks. Additionally, there is evidence that single bout [14-16] and lower intensity PA [17] have a positive effect on executive functions, impulsivity, response inhibition and performance in reading and arithmetic. However, most of these studies are small and uncontrolled. There is also a remarkable paucity of studies empirically investigating associations between the effects of PA and adult ADHD, despite the fact that PA has been linked to immediate and long term improvements in cognitive functioning in healthy adults [18,19]. BNDF, an important biomarker of the disorder, is significantly increased by regular physical exercise [20,21]. To our knowledge there is currently only one paper published on this subject (Pubmed research May 2014). Abramovitch et al. [22] found in an exploratory study, that frequent aerobic activity reduced intrusive thoughts, worry and impulsivity in a sample of 30 ADHD adults. According to anecdotal reports of patients with adult ADHD, PA can be a strategy to cope with ADHD symptoms. In some cases; however, frequency and intensity of the PA may reach an unhealthy level and become excessive. Excessive exercising should not per se be seen as a pathological form of behavior [23]. However, if individuals become preoccupied with exercising and if exercising is intensively time consuming resulting in the neglect of work, social life and health, exercise dependency (EXD) can be considered [24,25]. Excessive forms of exercising and ADHD have both been shown to occur more frequently in athletes [26-28]. Furthermore there is evidence that persons conducting excessive forms of physical activity commonly do so to suppress symptoms of anxiety, irritability, and restlessness [29,30].

**Table 1:** **Summary of studies investigating the effects of regular moderate-to-vigorous intensity exercise in children with ADHD**

**Study**	**Study design**	**Participants**	**Type of exercise**	**Key outcome measures**	**Findings**
Kang et al. 2011 [31]	RCT (exercise versus education for behavior control*)	n = 28	Twice a week during 6 consecutive weeks, moderate to vigorous intensity	Korean ADHD Rating Scale (K-ARS-PT), Digit Symbol and Trail-Making Test (TMT-B)	Greater improvement in attention symptoms, cognitive functioning and cooperativeness scores compared to control group.
		(28 male)			
		Mean age 8.6 years			
Chang et al. 2014 [32]	Controlled , non-randomized (exercise versus no exercise)	n = 27	Twice a week during 8 consecutive weeks, moderate intensity aquatic exercise	Basic Motor Ability Test Revised (BMAT), Go/NoGo Task	Greater improvements in accuracy associated with NoGo stimulus and coordination of motor skills compared to control group.
		(23 male)			
		5-10 years			
Verret et al. 2012 [33]	Controlled, non-randomized (exercise versus no exercise)	n = 21	3 times a week during 10 consecutive weeks, moderate to vigorous intensity	Child Behavior Checklist (CBCL), Test of Everyday Attention for Children (Tea-Ch)	Greater improvements in behavior reports by parents and teachers, information processing and auditory sustained attention compared to control group.
		(19 male)			
		7-12 years			
Smith et al. 2013 [34]	Open study,	n = 14	8 weeks of daily moderate-to-vigorous intensity	Broad range of measures including subtests from Wechsler preschool and Primary Scale of Intelligence (WPPSI-R), Wide Range Assessment of Memory and Learning (WRAML-2), Woodcock-Johnson III Test of Cognitive Abilities (WJ-III).	Largest Improvements in response inhibition / impulsiveness and behavior reports by parents, staff and teachers compared to pre-exercise levels.
	No control group	(6 male)			
		5-8 years			

*Additionally, methylphenidate treatment was newly established in both groups.

This is the first study empirically investigating the association between ADHD symptoms and excessive exercising in a representative population-based sample. Based on previous research and our own clinical experience, we hypothesized that individuals with positive ADHD screening results will be more likely to meet criteria of excessive exercising. A strong link between excessive forms of exercising and eating disorders (e.g. [35,36]) as well as anxiety and depression [37] have been found. Therefore information on anxiety, depression, and eating behavior was gathered to control for putative mediating variables.

## Methods

A representative sample of the German general population was selected with the assistance of a demographic consulting company (USUMA, Berlin, Germany). The sample was selected to be representative in terms of age, sex and education. A total of 2,520 people aged between 14 and 93 years agreed to participate and completed the self-rating questionnaires (participation rate: 61.9% of valid addresses attempted) between November 27 and December 16, 2009. A more detailed description of the procedure has been published in [11]. All respondents whose age was below 18 (n = 100) and above 64 years (n = 695) were excluded from the present study. There is concern about the accuracy of retrospective recall of childhood symptoms and about the validity of the self-rating instruments for childhood and current ADHD symptoms in older adults. In addition, participants who did not fully complete the central diagnostic instruments (WURS-k and ADHD-SR for ADHD and EDS-21 for excessive exercising) and those who did not provide weight data were excluded from further analyses. This resulted in a final sample of 1,615 individuals for analysis. The population-based survey met the ethical guidelines of the international Code of Marketing and Social Research Practice by the International Chamber of Commerce and the European Society for Opinion and Marketing Research. The survey was approved by the ethics committee of the University of Leipzig Medical School.

### Assessment

#### ADHD

Standard self-report screening instruments were used to assess childhood and adult ADHD. The German short version of the Wender Utah Rating Scale (WURS-k) [38] was used to retrospectively rate ADHD symptoms in childhood. The WURS-k consists of 21 items on a five-point Likert-scale (0–4, “not at all” to “severe”). We used a cut-off score of ≥ 30 to indicate the presence of a diagnosis of ADHD in childhood (age 8–10 years). This cut-off was proposed by the authors of the questionnaire with a sensitivity of 85% and a specificity of 76% for childhood ADHD [39]. The internal consistency in our sample was .88 (Cronbach’s α).

The ADHD self-rating scale (ADHD-SR) [40], was used to assess current ADHD symptoms. Based on the DSM-IV ADHD diagnostic criteria it includes 18 items to be rated on a four-point Likert-scale (0–3, “not at all” to “severe”). Subscale scores for inattention, hyperactivity, and impulsivity can be calculated. We used a cut-off score of ≥ 15 to indicate that participants met criteria for adult ADHD. This cut-off has been suggested by the authors of the questionnaire and has shown to exhibit a sensitivity of 77% and a specificity of 75% for adult ADHD [40]. The internal consistency in our sample was .92 (Cronbach’s α).

Both the WURS-k criteria and the ADHD-SR criteria had to be fulfilled for participants to be rated as likely cases of adult ADHD. We grouped the ADHD diagnoses into three mutually exclusive categories: never met diagnostic criteria (no ADHD), met full childhood criteria with no current symptoms (childhood only ADHD), and met full childhood criteria with current symptoms (adult ADHD).

#### Excessive exercising

The Exercise Dependence Scale (EDS-21) can be used to identify individuals performing excessive forms of exercising. Originally developed as a screening tool for exercise dependency [30,41], a total EDS score above 77 has previously been proposed as indicative for “exercise dependence at-risk” with a sensitivity of 1 and a specificity of 0.97 [42]. However, recently Müller et al. [23] have examined the concordance of the EDS with an interview-based diagnosis of EXD by assessing 134 subjects with both methods. They reported that the agreement between assessment methods is only fair to moderate with more false positive categorization by the EDS. They concluded that while the EDS does measure key factors of EXD, the clinical value of a high EDS score, however, remains debatable and probably points to the fact that the EDS not only measures exercise dependence traits but also other forms of “excessive exercising”. Similarly, Phelan et al. [43] conducted a study comparing PA and EXD between a group of successful weight-loss maintainers and a normal-weight control group also using the EDS-21. They found a significant difference between groups with significantly higher EDS-21 values in the weight-loss maintainer group. They concluded that this significant difference in EDS scores between groups appeared to reflect only subclinical symptoms of EXD and was more attributable to exercising for weight control than on what they called the “unhealthy” aspects of EXD. Considering the results of these 2 studies, a cutoff of score >77 was used to assess clinically relevant levels of excessive exercising for the purpose of the present study.

In the present study the German version (EDS-G) of the Exercise Dependence Scale was used which has been validated in a German general population sample [42]. The 21 items are rated on a six-point Likert scale ranging from 1 (never) to 6 (always) with three items per subscale. The total EDS-G scale shows an excellent internal reliability (Cronbach’s α in the present sample .97). Higher mean scores are positively related to a stronger incidence of excessive exercising and have additionally been linked to eating disorder symptoms.

#### Eating behavior

Typical aspects of eating-disorder related psychopathology were assessed using the German version of the Eating Disorder Examination Questionnaire (EDE-Q) [41,42]. The EDE-Q is a self-report questionnaire with good reliability and validity [44], German version [45] which is comprised of 4 subscales and a total score. For the purpose of this study only the EDE-Q total score was used. A global score threshold of ≥2.30 has been empirically derived [46] and was used to assess clinically relevant levels of eating disturbances. The internal consistency in our sample was .97 (Cronbach’s α). The BMI (kg/m^2^) was calculated based on the participants’ self-reported height and weight. A BMI ≥ 30 kg/m^2^ was recorded as obesity.

#### Depression and anxiety

The German version of the Patient Health Questionnaire (PHQ-4), an ultra-brief 4-item self-report questionnaire, was used as an overall screening tool for depression and anxiety [47], German version [48]. It contains a 2-item depression scale (PHQ-2 [49]) and a 2-item anxiety scale (GAD-2 [50]). The symptoms are assessed for the last 2 weeks and for each of the four questions the response options range from 0 (‘not at all’) to 3 (‘nearly every day’). The subscales PHQ-2 and GAD-2 are highly intercorrelated (r = 0.61) and the authors of the scale therefore recommended considering the PHQ-4 total scale as an overall depression and anxiety screening tool. For the analyses a dichotomous variable with a cut-off of 6 and above was used. This cut-off has been suggested to screen for the presence of a depressive or an anxiety disorder, representing a percentile of 95.7% in a large population based sample (N = 5,030) [47].

### Statistics

All statistical analyses were conducted using the statistical package for social sciences (SPSS V21) for Windows. Individuals without ADHD (noADHD), with childhood only ADHD (coADHD) and with adult ADHD (aADHD) (3 categories) were compared on several variables using chi square tests and pair-wise contrasts with chi-square tests between two groups for categorical variables as well as ANOVAs with Tukey B post-hoc tests for continuous variables.

Multinomial logistic regression analyses were conducted with ADHD categories as the dependent variable and excessive exercising as the main independent variable. Regression analyses were carried out in 3 steps: without adjustment, controlling for socio-demographic variables (sex, age, and educational level and employment status), and controlling for socio-demographic variables as well as BMI, EDE-Q, and PHQ-4 cutoff scores. An alpha-level of 0.05 was adopted for all tests.

## Results

A description of the total study sample (n = 1,615) and by groups is given in Table 2. The mean age of the sample was 43.3 (SD 12.7) years. There was a significant difference between the noADHD and the coADHD groups with coADHD individuals being significantly younger (Table 3). Of the total sample 53.7% were female, 8.6% were unemployed, and 15.8% had finished high school or attained education beyond high school. Screening for current depression and anxiety with the PHQ-4 (cut-off ≥ 6) revealed positive results in 5.7% of the sample; 4.6% of the sample screened positive for adult ADHD with a persistence of ADHD-symptoms from childhood into adulthood of 41.6%; 3.1% (n = 51) screened positive for excessive exercising. The prevalence of depression/anxiety and eating disorders, as well as the distribution of gender, weight, employment, educational, and marital status among noADHD, coADHD and aADHD subgroups are in line with earlier literature reports [11,51,52] (Table 3 and 2)*.*

**Table 2:** **Sociodemographic characteristics, PHQ-4, EDE-Q and EDS-G results for the total sample and the ADHD subgroups**

	**Total**	**noADHD**	**coADHD**	**aADHD**	**χ** ^**2**^
	**(n = 1615)**	**(n = 1451)**	**(n = 89)**	**(n = 75)**	**(df = 2)**
Female	53.7	54.9^a^	32.6^b^	54.7^a^	16.9; p < 0.001
Never married	27.5	26.1^a^	42.7^b^	37.3^b^	15.5; p < 0.001
Unemployed	8.6	7.9^a^	7.9^a^	22.7^b^	19.8; p < 0.001
High school or beyond	15.8	16.5^a^	14.6^a^	2.7^b^	10.4; p = 0.005
Obesity	10.7	10.5^a^	5.6^a^	21.3^b^	11.3; p < 0.005
PHQ-4 ≥ 6	5.7	4.5^a^	6.7^a^	28.0^b^	73.6; p < 0.001
EDE-Q ≥ 2.30	4.4	3.7^a^	4.5^a^	17.3^b^	31.4; p < 0.001
EDS-G > 77	3.1	2.7^a^	9.0^b^	4.0^ab^	11.3; p < 0.005

Data are shown as percentages. Values with different superscripts are significantly different (pair-wise contrasts with chi-square tests between two groups).

aADHD = adult ADHD; coADHD = childhood only ADHD; noADHD = no ADHD.

PHQ-4 = Patient Health Questionnaire; EDS-G = Exercise Dependence Scale; EDE-Q = Eating Disorder Examination Questionnaire.

**Table 3:** **Age and questionnaire results**

	**Total**	**noADHD**	**coADHD**	**aADHD**	**ANOVA, F**
	**(n = 1615)**	**(n = 1451)**	**(n = 89)**	**(n = 75)**	**(df = 2)**
Age	43.3 (12.7)	43.6 (12.6)^a^	40.1 (12.5)^b^	40.7 (13.6)^ab^	4.907
					p = 0.008
PHQ-4 total score	1.5 (2.1)	1.3 (2.0)^a^	1.5 (2.1)^a^	4.1 (3.2)^b^	66.779
					p < 0.001
EDE-Q total score	0.5 (0.8)	0.5 (0.7)^a^	0.5 (0.8)^a^	1.0 (1.1)^b^	16,754
					p < 0.001
EDS-G total score	32.1 (16.8)	31.5 (16.2)^a^	37.5 (22.8)^b^	37.0 (19.5)^b^	8.748
					p < 0.001

Data are shown as mean and SD. Values with different superscripts are significantly different (Tukey B post-hoc tests).

aADHD = adult ADHD; coADHD = childhood only ADHD; noADHD = no ADHD.

PHQ-4 = Patient Health Questionnaire; EDS-G = Exercise Dependence Scale; EDE-Q = Eating Disorder Examination Questionnaire.

aADHD (37.0) and coADHD (37.5) both showed significantly higher mean scores on the EDS-G compared to the noADHD group (31.5); however, these values are rather low and they are close to the range of the norm population [42]. Mean scores among eating disorder patients or clients of fitness centers have been shown to be approximately twice as high [23]. Therefore a cutoff >77 on the EDS-G was used to define values in a putative pathological range and assess clinically relevant levels of excessive exercising. Individuals with coADHD showed a significantly higher frequency of excessive exercising (9.0%) than individuals without ADHD (2.7%) (χ^2^ = 11.252, df = 1, p = 0.001). There was no difference between individuals with coADHD and aADHD (4%) (χ^2^ = 1.619, df = 1, p = 0.2) and between individuals without ADHD and with aADHD (χ^2^ = 0.459, df = 1, p = 0.5) in the frequency of excessive exercising (Table 2).

Multinomial logistic regression analyses revealed that a mean score on the EDS-G above the proposed cutoff for excessive exercising was significantly associated with coADHD compared to noADHD with an odds ratio of 3.239 even after controlling for socio-demographic variables, BMI, eating related (EDE-Q) and general psychopathology (PHQ-4). No significant association could be found between excessive exercising and aADHD (Table 4).

**Table 4:** **Multinomial logistic regression analyses**

**EXD (>77 EDS-G)**	**coADHD (vs. noADHD)**	**aADHD (vs. noADHD)**
	**odds ratio (95% CI)**	**odds ratio (95% CI)**
	**B, standard error, Wald**	**B, standard error, Wald**
Unadjusted		
No	1.0	1.0
Yes	3.576 (1.618-7.903)**	1.509 (0.455-4.998)
	1.274, .405, 9.918	.411, .611, .453
Adjusted for SDV		
No	1.0	1.0
Yes	3.199 (1.340-7.638)**	0.693 (0.091-5.275)
	1.163, .444, 6.861	-.366, 1.035, .125
Adjusted for SDV,		
PHQ-4, EDE-Q, BMI		
No	1.0	1.0
Yes	3.239 (1.329-7.896)**	0.619 (0.077-4.960)
	1.175, .455, 6.681	-.480, 1.062, .204

**p ≤ 0.01.

Sociodemographic variables (SDV) = Sex, age, educational level, employment status.

aADHD = adult ADHD; coADHD = childhood only ADHD; noADHD = no ADHD.

PHQ-4 = Patient Health Questionnaire; EDS-G = Exercise Dependence Scale; EDE-Q = Eating Disorder Examination Questionnaire.

Likelihood ratio tests for the models:

Unadjusted: χ^2^ = 7.920, df = 2, p = 0.01.

Adjusted for SDV: χ^2^ = 50.846, df = 10, p < .001.

Adjusted for SDV, PHQ-4, EDE-Q, BMI: χ^2^ = 88.709, df = 16, p < .001.

## Discussion

The primary purpose of the present investigation was to analyze the association of ADHD symptoms with symptoms of excessive exercising in an adult general population sample. We had expected to find that individuals with positive ADHD screening are more likely to meet criteria for excessive exercising. In summary, our results show that this only applies to the subgroup of participants with childhood only ADHD. The point prevalence of excessive exercising at risk was estimated to be 9% in individuals with childhood only ADHD as opposed to individuals without ADHD (2.7%) and individuals with adult ADHD (4%). The odds ratio for excessive exercising in the childhood only ADHD group was 3.2 compared to the no ADHD group even after adjusting for putative mediating variables.

A first concern may be that the ADHD-SR and EDS-G scales both measure excessive physical activity and associations between results of these questionnaires may therefore be due to an overlap of the questionnaire items. The authors have thoroughly screened and compared the items of the questionnaires and found an only trivial, negligible overlap concerning the measurement of physical activity (face validity) (e.g. ADHS-SR: “Item 1: I am inattentive to details or lack diligence at work.”; best match EDS-G: “Item 12: I think about exercise when I should be concentrating on school.”). We also found the prevalence rates of adult ADHD symptoms, their persistence into adulthood and of excessive exercising or “at-risk” EXD in the present study to be in line with earlier literature reports [6,8,53,54].

There are several possible interpretations of the results. It is well known that ADHD has an increased comorbidity with a range of psychiatric disorders such as conduct disorder, mood disorders, anxiety disorders, and substance dependence [55-57]. The presumption that the association between coADHD and an EDS-G score >77 is a hint towards an increased prevalence of exercise dependency as another comorbidity in individuals with ADHD, therefore, might seem legitimate at first glance. However this could not satisfactorily explain that such correlation cannot be found in the adult ADHD group where it would then be most expected to manifest itself.

This implies that a proportion of coADHD individuals might suppress their ADHD symptoms by excessive sporting activities. Excessive exercising could thus be one possible factor to cope with ADHD symptoms in adulthood. Recent studies have focused on the effects of physical activity on ADHD symptoms in children (e.g. [13]) and the proposed underlying mechanisms might equally be applicable to ADHD in adulthood [21]. Using a meta-regression analysis, Lambourne et al. [58] showed that regardless of the type of exercise cognitive task performance improved after physical activity.

Bäckmann et al. [59] have provided a theoretical framework for psychological compensation. They defined compensation as a change in the behavioral profile of an individual to counterbalance a mismatch between accessible skills and environmental demands. Three main dimensions were proposed: counterbalancing a cognitive deficit by increased time or effort, use of latent skills or acquisition of new skills. Concerning increased time and effort, Merkt et al. [60] have shown that individuals with ADHD compensate by trading of speed for increased accuracy when performing a task requiring a high degree of focused attention. According to these studies, excessive exercising could be interpreted as the activation of a latent skill to decrease symptoms of ADHD. It remains uncertain however whether this is based on awareness of ADHD related deficits and reflects a deliberate decision to compensate or whether an automatic compensation response has been initiated. Although compensatory behavior is usually linked to awareness and deliberation, compensation for some, especially innate, handicaps can be a subconscious process [61].

It is assumed that compensatory behavior to increase functioning in a selected domain is carried out at the expense of functioning in other areas [61] and that consequences of compensatory behavior range along a continuum of positive to maladaptive outcomes, which may explain negative impacts associated with excessive forms of exercising (e.g. physical injury, etc.).

On a neurobiological level, research suggests that PA leads to increased cerebral blood flow in animal models [62] as well as improved cerebral structures and increased brain activity in regions associated with attention control processes in humans [19]. There is also evidence that it might promote brain plasticity [63,64]. Assuming a catecholamine metabolism defect as a key factor in ADHD pathogenesis, Wigal et al. [65] speculate that exercise induced alteration of the catecholamine release affects similar noradrenergic and dopaminergic systems that stimulant medications aim at and could therefore play an important role in modulating ADHD symptoms. The increased sporting activity of the coADHD individuals in our sample could therefore possibly be interpreted in terms of a (successful) self-treatment, alleviating ADHD symptoms in adults to a sub-clinical level. This could also be an explanation why ADHD has been shown to occur more frequently in athletes [26,27]. In a currently published literature review, White et al. [66] concluded that exercise may benefit many athletes with ADHD.

The strengths of the present study include the use of standardized questionnaires in a large sample of the general population. By avoiding a self-selected sample or a clinical ADHD cohort, individuals outside of treatment settings and without diagnosed ADHD were acquired. Though, due to the use of screening questionnaires no confirmed diagnoses could be made. With regard to the aADHD sample, sociodemographic variables such as the relatively high proportion of unemployed individuals and the rather low education status seem quite typical for adults with ADHD. Similarly the low rate of female gender among coADHD and the equal balance between men and women among aADHD, is well in line with earlier literature reports [67]. Nevertheless, the definition of different groups based solely on self-ratings can be viewed as a possible shortcoming of the investigation. However it must be taken into account, that it seems illusionary to apply structured interviews in a general population sample of this size. Another limitation is that current medications or other therapies possibly applied were not recorded. Because the focus was set on an excessive form of PA, there was no information gathered about regular “healthy” physical activity of the individuals. Because this study is partially retrospective the possibility that there is an unknown confounder that might contribute to both a history of ADHD and excessive exercise (rather than excessive exercise causally reducing ADHD symptoms) cannot be ruled out. Further research is therefore encouraged to examine the effect of physical activity on ADHD symptoms in adults. Ideally this would be a case–control study using a clinical ADHD cohort and questionnaires to record normal exercise behavior. The age of onset and extend of excessive exercising behavior should be recorded to allow for more accurate assessment of the self-treatment hypothesis in further studies. Furthermore, controlled studies exploring the potential of PA in the treatment of ADHD in adulthood are warranted.

## Conclusions

This study is the first to investigate the association between adult ADHD and excessive exercising. We found that individuals with childhood only ADHD demonstrated a higher prevalence of excessive exercising, which might help to suppress ADHD symptoms in adulthood in some individuals. Even though the causality of this effect is supported by previous literature it cannot be confirmed due to the applied study design. However, if exercise therapy proves to have a benefit in ADHD patients, this would have important public health implications. This type of therapy is likely to be cost-effective, could be readily disseminated and would have a beneficial influence on many co-occurring psychiatric and non-psychiatric diseases. Further research is therefore encouraged.

## References

[1] American Psychiatric Association (2013). Diagnostic and Statistical Manual of Mental Disorders: 5th ed.

[2] Klein RG, Mannuzza S, Olazagasti MA, Roizen E, Hutchison JA, Lashua EC, Castellanos FX (2012). Clinical and functional outcome of childhood attention-deficit/hyperactivity disorder 33 years later. Arch Gen Psychiatry.

[3] Willcutt EG, Doyle AE, Nigg JT, Faraone SV, Pennington BF (2005). Validity of the executive function theory of attention-deficit/hyperactivity disorder: a meta-analytic review. Biol Psychiatry.

[4] Shallice T, Marzocchi GM, Coser S, Del Savio M, Meuter RF, Rumiati RI (2002). Executive function profile of children with attention deficit hyperactivity disorder. Dev Neuropsychol.

[5] Yamada K, Nabeshima T (2003). Brain-derived neurotrophic factor/TrkB signaling in memory processes. J Pharmacol Sci.

[6] Faraone SV, Biederman J, Mick E (2006). The age-dependent decline of attention deficit hyperactivity disorder: a meta-analysis of follow-up studies. Psychol Med.

[7] Barkley RA, Fischer M, Smallish L, Fletcher K (2002). The persistence of attention-deficit/hyperactivity disorder into young adulthood as a function of reporting source and definition of disorder. J Abnorm Psychol.

[8] de Zwaan M, Gruss B, Müller A, Graap H, Martin A, Glaesmer H, Hilbert A, Philipsen A (2012). The estimated prevalence and correlates of adult ADHD in a German community sample. Eur Arch Psychiatry Clin Neurosci.

[9] Polanczyk G, Rohde LA (2007). Epidemiology of attention-deficit/hyperactivity disorder across the lifespan. Curr Opin Psychiatry.

[10] Biederman J, Monuteaux MC, Mick E, Spencer T, Wilens TE, Silva JM, Snyder LE, Faraone SV (2006). Young adult outcome of attention deficit hyperactivity disorder: a controlled 10-year follow-up study. Psychol Med.

[11] de Zwaan M, Gruss B, Müller A, Philipsen A, Graap H, Martin A, Glaesmer H, Hilbert A (2011). Association between obesity and adult attention-deficit/hyperactivity disorder in a German community-based sample. Obes Facts.

[12] Grassmann V, Alves MV, Santos-Galduroz RF, Galduroz JC: **Possible cognitive benefits of acute physical exercise in children with ADHD: a systematic review.** *J Atten Disord* 2014, in press (doi:10.1177/1087054714526041). 10.1177/1087054714526041 24621460

[13] Gapin JI, Labban JD, Etnier JL (2011). The effects of physical activity on attention deficit hyperactivity disorder symptoms: the evidence. Prev Med.

[14] Pontifex MB, Saliba BJ, Raine LB, Picchietti DL, Hillman CH (2013). Exercise improves behavioral, neurocognitive, and scholastic performance in children with attention-deficit/hyperactivity disorder. J Pediatr.

[15] Medina JA, Netto TL, Muszkat M, Medina AC, Botter D, Orbetelli R, Scaramuzza LF, Sinnes EG, Vilela M, Miranda MC (2010). Exercise impact on sustained attention of ADHD children, methylphenidate effects. Atten Defic Hyperact Disord.

[16] Chang YK, Liu S, Yu HH, Lee YH (2012). Effect of acute exercise on executive function in children with attention deficit hyperactivity disorder. Arch Clin Neuropsychol.

[17] Kamp CF, Sperlich B, Holmberg HC (2014). Exercise reduces the symptoms of attention-deficit/hyperactivity disorder and improves social behaviour, motor skills, strength and neuropsychological parameters. Acta Paediatr.

[18] Winter B, Breitenstein C, Mooren FC, Voelker K, Fobker M, Lechtermann A, Krueger K, Fromme A, Korsukewitz C, Floel A, Knecht S (2007). High impact running improves learning. Neurobiol Learn Mem.

[19] Colcombe S, Kramer AF (2003). Fitness effects on the cognitive function of older adults: a meta-analytic study. Psychol Sci.

[20] Erickson KI, Voss MW, Prakash RS, Basak C, Szabo A, Chaddock L, Kim JS, Heo S, Alves H, White SM, Wojcicki TR, Mailey E, Vieira VJ, Martin SA, Pence BD, Woods JA, McAuley E, Kramer AF (2011). Exercise training increases size of hippocampus and improves memory. Proc Natl Acad Sci U S A.

[21] Archer T, Kostrzewa RM (2012). Physical exercise alleviates ADHD symptoms: regional deficits and development trajectory. Neurotox Res.

[22] Abramovitch A, Goldzweig G, Schweiger A (2013). Correlates of physical activity with intrusive thoughts, worry and impulsivity in adults with attention deficit/hyperactivity disorder: a cross-sectional pilot study. Isr J Psychiatry Relat Sci.

[23] Müller A, Cook B, Zander H, Herberg A, Müller V, de Zwaan M (2014). Does the German version of the exercise dependence scale measure exercise dependence?. Psychol Sport Exerc.

[24] de Coverley Veale DM (1987). Exercise dependence. Br J Addict.

[25] Hausenblas HA, Symons Downs D (2002). Exercise dependence: a systematic review. Psychol Sport Exerc.

[26] Parr JW (2011). Attention-deficit hyperactivity disorder and the athlete: new advances and understanding. Clin Sports Med.

[27] Putukian M, Kreher JB, Coppel DB, Glazer JL, McKeag DB, White RD (2011). Attention deficit hyperactivity disorder and the athlete: an American medical society for sports medicine position statement. Clin J Sport Med.

[28] Allegre B, Therme P, Griffiths M (2007). Individual factors and the context of physical activity in exercise dependence: a prospective study of “Ultra-Marathoners”. Int J Ment Health Addiction.

[29] Aidman EV, Woollard S (2003). The influence of self-reported exercise addiction on acute emotional and physiological responses to brief exercise deprivation. Psychol Sport Exerc.

[30] Hausenblas HA, Downs DS (2002). How much is too much? The development and validation of the exercise dependence scale. Psychol Health.

[31] Kang KD, Choi JW, Kang SG, Han DH (2011). Sports therapy for attention, cognitions and sociality. Int J Sports Med.

[32] Chang YK, Hung CL, Huang CJ, Hatfield BD, Hung TM (2014). Effects of an aquatic exercise program on inhibitory control in children with ADHD: a preliminary study. Arch Clin Neuropsychol.

[33] Verret C, Guay MC, Berthiaume C, Gardiner P, Beliveau L (2012). A physical activity program improves behavior and cognitive functions in children with ADHD: an exploratory study. J Atten Disord.

[34] Smith AL, Hoza B, Linnea K, McQuade JD, Tomb M, Vaughn AJ, Shoulberg EK, Hook H (2013). Pilot physical activity intervention reduces severity of ADHD symptoms in young children. J Atten Disord.

[35] Cook BJ, Hausenblas HA (2008). The role of exercise dependence for the relationship between exercise behavior and eating pathology: mediator or moderator?. J Health Psychol.

[36] Cook B, Engel S, Crosby R, Hausenblas H, Wonderlich S, Mitchell J (2014). Pathological motivations for exercise and eating disorder specific health-related quality of life. Int J Eat Disord.

[37] Costa S, Hausenblas HA, Oliva P, Cuzzocrea F, Larcan R (2013). The role of age, gender, mood states and exercise frequency on exercise dependence. J Behav Addictions.

[38] Retz-Junginger P, Retz W, Blocher D, Weijers HG, Trott GE, Wender PH, Rossler M (2002). Wender Utah rating scale. The short-version for the assessment of the attention-deficit hyperactivity disorder in adults. Nervenarzt.

[39] Retz-Junginger P, Retz W, Blocher D, Stieglitz RD, Georg T, Supprian T, Wender PH, Rosler M (2003). Reliability and validity of the Wender-Utah-rating-scale short form. Retrospective assessment of symptoms for attention deficit/hyperactivity disorder. Nervenarzt.

[40] Rosler M, Retz W, Retz-Junginger P, Thome J, Supprian T, Nissen T, Stieglitz RD, Blocher D, Hengesch G, Trott GE (2004). Tools for the diagnosis of attention-deficit/hyperactivity disorder in adults. Self-rating behaviour questionnaire and diagnostic checklist. Nervenarzt.

[41] Downs DS, Hausenblas HA, Nigg CR (2004). Factorial validity and psychometric examination of the exercise dependence scale-revised. Meas Phys Educ Exerc Sci.

[42] Müller A, Claes L, Smits D, Gefeller O, Hilbert A, Herberg A, Müller V, Hofmeister D, de Zwaan M (2013). Validation of the German version of the exercise dependence scale. Eur J Psychol Assess.

[43] Phelan S, Bond DS, Lang W, Jordan D (2011). Wing RR: “Exercise dependence”–a problem or natural result of high activity?. Am J Health Behav.

[44] Fairburn CG, Beglin SJ (1994). Assessment of eating disorders: interview or self-report questionnaire?. Int J Eat Disord.

[45] Hilbert A, Tuschen-Caffier B, Karwautz A, Niederhofer H, Munsch S (2007). Eating disorder examination-questionnaire: psychometric properties of the German version. Diagnostica.

[46] Mond JM, Hay PJ, Rodgers B, Owen C, Beumont PJ (2004). Validity of the eating disorder examination questionnaire (EDE-Q) in screening for eating disorders in community samples. Behav Res Ther.

[47] Kroenke K, Spitzer RL, Williams JB, Lowe B (2009). An ultra-brief screening scale for anxiety and depression: the PHQ-4. Psychosomatics.

[48] Lowe B, Wahl I, Rose M, Spitzer C, Glaesmer H, Wingenfeld K, Schneider A, Brahler E (2010). A 4-item measure of depression and anxiety: validation and standardization of the patient health questionnaire-4 (PHQ-4) in the general population. J Affect Disord.

[49] Kroenke K, Spitzer RL, Williams JB (2003). The patient health questionnaire-2: validity of a two-item depression screener. Med Care.

[50] Kroenke K, Spitzer RL, Williams JB, Monahan PO, Lowe B (2007). Anxiety disorders in primary care: prevalence, impairment, comorbidity, and detection. Ann Intern Med.

[51] Rucklidge JJ, Downs-Woolley M, Taylor M, Brown JA, Harrow SE: **Psychiatric comorbidities in a New Zealand sample of adults with ADHD.** *J Atten Disord* 2014, in press (doi:10.1177/1087054714529457). 10.1177/1087054714529457 24743977

[52] Harpin VA (2005). The effect of ADHD on the life of an individual, their family, and community from preschool to adult life. Arch Dis Child.

[53] Kessler RC, Adler L, Barkley R, Biederman J, Conners CK, Demler O, Faraone SV, Greenhill LL, Howes MJ, Secnik K, Spencer T, Ustun TB, Walters EE, Zaslavsky AM (2006). The prevalence and correlates of adult ADHD in the United States: results from the National Comorbidity Survey Replication. Am J Psychiatry.

[54] Sussman S, Lisha N, Griffiths M (2011). Prevalence of the addictions: a problem of the majority or the minority?. Eval Health Prof.

[55] Bauermeister JJ, Shrout PE, Ramirez R, Bravo M, Alegria M, Martinez-Taboas A, Chavez L, Rubio-Stipec M, Garcia P, Ribera JC, Canino G (2007). ADHD correlates, comorbidity, and impairment in community and treated samples of children and adolescents. J Abnorm Child Psychol.

[56] Lee SS, Humphreys KL, Flory K, Liu R, Glass K (2011). Prospective association of childhood attention-deficit/hyperactivity disorder (ADHD) and substance use and abuse/dependence: a meta-analytic review. Clin Psychol Rev.

[57] Biederman J, Newcorn J, Sprich S (1991). Comorbidity of attention deficit hyperactivity disorder with conduct, depressive, anxiety, and other disorders. Am J Psychiatry.

[58] Lambourne K, Tomporowski P (2010). The effect of exercise-induced arousal on cognitive task performance: a meta-regression analysis. Brain Res.

[59] Bäckman L, Dixon RA (1992). Psychological compensation: a theoretical framework. Psychol Bull.

[60] Merkt J, Singmann H, Bodenburg S, Goossens-Merkt H, Kappes A, Wendt M, Gawrilow C (2013). Flanker performance in female college students with ADHD: a diffusion model analysis. Atten Defic Hyperact Disord.

[61] Baltes PB, Staudinger UM, Lindenberger U (1999). Lifespan psychology: theory and application to intellectual functioning. Annu Rev Psychol.

[62] Endres M, Gertz K, Lindauer U, Katchanov J, Schultze J, Schrock H, Nickenig G, Kuschinsky W, Dirnagl U, Laufs U (2003). Mechanisms of stroke protection by physical activity. Ann Neurol.

[63] Cotman CW, Berchtold NC (2002). Exercise: a behavioral intervention to enhance brain health and plasticity. Trends Neurosci.

[64] Smith AE, Goldsworthy MR, Garside T, Wood FM, Ridding MC (2014). The influence of a single bout of aerobic exercise on short-interval intracortical excitability. Exp Brain Res.

[65] Wigal SB, Emmerson N, Gehricke JG, Galassetti P (2013). Exercise: applications to childhood ADHD. J Atten Disord.

[66] White RD, Harris GD, Gibson ME (2014). Attention deficit hyperactivity disorder and athletes. Sports Health.

[67] Bauermeister JJ, Shrout PE, Chavez L, Rubio-Stipec M, Ramirez R, Padilla L, Anderson A, Garcia P, Canino G (2007). ADHD and gender: are risks and sequela of ADHD the same for boys and girls?. J Child Psychol Psychiatry.

